# Embedding the patient voice into research on spontaneous preterm birth—themes from a Preterm Birth Advisory Council

**DOI:** 10.1371/journal.pone.0312370

**Published:** 2024-12-20

**Authors:** Gillian Corbett, Mandy Daly, Dylan Keegan, Patricia Horgan, Catriona Keyes, Larissa Luethe, Siobhan Corcoran, Fionnuala M. McAuliffe

**Affiliations:** 1 UCD Perinatal Research Centre, UCD School of Medicine, University College Dublin, National Maternity Hospital, Dublin, Ireland; 2 National Maternity Hospital, Dublin, Ireland; 3 Preterm Birth Advisory Council, National Maternity Hospital, Dublin, Ireland; 4 Irish Neonatal Health Alliance, Bray, Co. Wicklow, Ireland; 5 UCD School of Medicine, UCD Clinical Research Centre, University College Dublin, Dublin, Ireland; University of the Witwatersrand, SOUTH AFRICA

## Abstract

**Background:**

Spontaneous preterm birth (sPTB) has a deep immediate impact on patients but also alters their care and experience in subsequent pregnancies. There is an absence of the pregnant patient’s voice in the research surrounding pregnancy at risk of sPTB.

**Materials/methods:**

The Preterm Birth Advisory Council was established at the National Maternity Hospital (NMH) in January 2023, to introduce and embed the patient voice in research into sPTB prevention. Council members include patients with lived experience of sPTB, patient advocate representatives and clinicians involved in sPTB preventative care. Topics around sPTB prevention were openly discussed with experts by experience and shared with sPTB advocacy groups. Responses were analysed for themes most important to those with lived experience. Ethical approval was granted by NMH Research Ethics Committee.

**Results:**

In total, seven experts by experience gave their views over the course of a three month period. Six key themes were observed:

1. Clinical Outcomes in Preterm Birth Research. Low priority was placed on the modality, route and duration of interventions. The most important aspect of an intervention is its clinical efficacy. High tolerance thresholds were described in sacrifice for a positive pregnancy outcome
2. Preterm Birth Preventative care as a patient-led care model. While medical and surgical interventions play their role in objectively reducing risk, much of pregnancy care after preterm birth centres on psychological support and continuity of care.
3. Lack of awareness on risk factors for sPTB is an obstacle to timely referral and access to care. Patient experience of preterm birth prevention and timely interventions are improved where risk factor awareness exists.
4. Importance of preconceptual counselling. The unexpected nature of adverse outcome adds additional trauma to the event itself. Anticipation of adverse event can improve sense of control, minimise trauma and increase ability to cope.
5. The partner’s experience of pregnancy at risk of preterm birth is largely missing from focus of research and clinical care. Peer-support, digital resources or partner handbooks may be helpful for support persons.
6. Traumatic language included referring to a mid-trimester pregnancy loss or peri-viable preterm birth as a ‘miscarriage.’ Regarding language around preterm birth, there was no preferred term identified for women with risk factors or experience of preterm birth.

**Conclusions:**

The Preterm Birth Advisory Council places the voice of those impacted by preterm birth at the centre of research into its prevention. The themes identified may guide activities within this research area in local settings and international platforms. It is the council’s hope that supported by their work, it will be the patient’s voice that rings loudest in research in spontaneous preterm birth prevention.

## Introduction

The experience of spontaneous preterm birth (sPTB) is extremely common, affecting one in twenty pregnancies in Western countries and up to one in ten in under-resourced environments. Although harm from preterm birth is felt by the whole family, the parental burden of preterm birth is borne disproportionately by the mother [1, 2]. Despite this, there is a relative absence of maternal voice in sPTB prevention when compared to the focus on strategies for prevention. Inclusion of the patient’s voice has improved care pathways in many conditions, including placenta accreta syndrome (PAS) [3], post-pregnancy cohorts [4] and young persons’ groups [5]. Involvement of public and patient involvement (PPI) groups can greatly strengthen delivery of care and clinical research [6], with the key stakeholder, the patient, being placed at the centre of the conversation.

Some recent groups have captured the maternal voice in relation to specific sPTB interventions. Shennan et al’s group recently evaluated the impact of trans-abdominal cerclage for women experiencing recurrent preterm birth. Using qualitative surveys, they analysed women’s views on the intervention, who framed it as a vehicle for hope for those who have given up on parenthood [7], a powerful message for families everywhere experiencing recurrent spontaneous preterm birth or pregnancy loss. Other groups have illustrated how parental perspective can identify core outcome sets that carry significance for patients in PTB and other research [4, 8], using clinical stake holder qualitative interviews to analyse views. However, partnership between clinicians in preterm birth and families themselves is an exciting emerging concept that holds vast potential for care and research enhancement.

The experience of the patient in pregnancy at risk of spontaneous preterm birth has been shown to have a direct impact on outcomes [9]. Women’s experience of preterm birth can significantly affect their family plans and subsequent pregnancies [10]. This alone highlights the importance of the maternal experience in care around preterm birth and its prevention. With this in mind, the Preterm Birth Advisory Council was founded in Ireland in 2023. This manuscript describes the discussion themes of this patient-led advisory council. We also present the Council’s suggestions to enhance care for families experiencing spontaneous preterm birth and embed the maternal voice into preterm birth prevention research.

## Materials and methods

This is a report of the founding of a Preterm Birth Advisory Council and its initial findings from focused discussions from April to September 2023.

### The Preterm Birth Advisory Council

The Preterm Birth Advisory Council (PBAC) was established at the National Maternity Hospital Dublin Ireland in January 2023, to embed the patient voice into the arena of preterm birth research. It was noted that many patients and prior research participants within the preterm birth service had expressed interest in contribution to research directions, and had very helpful suggestions on how to improve care pathways. From this feedback, the concept of the advisory council was jointly founded between patients and clinicians. Council founding members include patients with lived experience of preterm birth (n = 5), patient advocate representatives (n = 1), clinicians involved in preterm birth prevention (n = 4). The aim of the group was to understand key outcomes of importance to women in preterm birth prevention research, to seek advice related to how the hospital’s research agenda could be more relevant to the patient’s needs and establish a pathway for patient-led improvements in clinical care. The Council is independent but complimentary to the Preterm Birth Prevention Service at the National Maternity Hospital, Dublin, Ireland.

### Setting up the NMH Preterm Birth Advisory Council

Co-founding clinicians members were those involved in delivery of care at the preterm birth service at the National Maternity Hospital. These clinicians have a research partnership with the UCD Clinical Research Centre, who’s patient advocate representative was approached to join the council and standardise the quality of PPI integration. This representative has extensive experience in PPI activities, liaising with families and integrating with the national PPI Network and the PPI Ignite Network at University College Dublin. Initial patient representatives were identified by researchers based on their reputation of advocacy in the experience of preterm birth. Patient co-founding members were those who had experienced interest in advocacy work and contribution to clinical care improvements and research within the department. All patients members involved either had a history of prior preterm birth or mid-trimester loss. Some had attended a preterm birth prevention clinic in a subsequent pregnancy. All co-founding members submitted Expression of Interest forms which included statements of motivation to join the council and written consent forms to participate. Expression of interest forms detailed the goal of the council, the roles and responsibility of members, details on meetings timing and information on selection process and data protection. Terms of Reference were drafted with input from patient representatives.

### Meetings of the NMH Preterm Birth Advisory Council

Meetings were conducted from April 2023 to Nov 2023 using the online telecommunications platform Zoom™ (Zoom™ Video Communications Inc., California, United States of America). This was considered the most appropriate interface for ensuring participant safety and confidentiality. Each meeting lasted approximately one hour. The content and delivery of the meetings was devised using training provided by the UCD Clinical Research Centre with integration of the Patient Advocate Representative to the meeting and their involvement in preparation. The primary focus of the initial meeting was to create familiarity between all group members, establish common understanding of the focus of the group and become familiar with the individual skillsets of all stakeholders. Clinical action points were identified at the close of each meeting and summaries of meeting notes were shared thereafter.

The focus of subsequent discussion was the experience of pregnancy care for those at high-risk of spontaneous preterm birth. Topics around preterm birth prevention were discussed, and patients were encouraged to freely express views. If encountered, the distress protocol adopted was the Qualitative Research Distress Protocol, where participants were followed up and appropriate support was facilitated [11]. Meetings were recorded and transcripts generated by the telecommunications software. Response and discussion transcripts were then analysed using reflexive thematic analysis to identify themes using codes, descriptions and patterns. Reflexive thematic Analysis method [12] was used to do this, which involved several steps: familiarization with data content, generation of initial codes, searching for themes, reviewing themes, confirming theme names and finally producing the report. Statements were grouped based on shared codes and potential themes. Lists of grouped statements were then reviewed to examine for emerging patterns. Key themes were identified. Data were saturated and no new themes or patterns emerged after responses six and seven.

Ethical approval was obtained to establish the Preterm Birth Advisory Council from the National Maternity Hospital Research Ethics Committee in March 2023 (Study ID: EC37.2020). Informed written consent to participant in Advisory Council was obtained via the expression of interest forms.

## Results

The outputs of the first four meetings were used to capture themes. Meetings were semi-structured around several discussion points. In total, seven experts by experience gave their views over the course of these meetings. All seven had a history of prior preterm birth or mid-trimester loss, with or without history of cervical surgery. All were of European ancestry and 34–35 years of age at time of spontaneous preterm birth between (34–35 years), while current age is 39–54 years of age. Gestational age at prior spontaneous preterm birth or mid-pregnancy loss varied from 18 to 33 weeks. For clinical members, experience of care around spontaneous preterm birth ranged from eight to thirty years. Experience in patient advocacy varied between one and eight years.

Six themes were observed and are summarised in Table 1.

**Table 1:** Key themes and subthemes in research into spontaneous preterm birth identified at the Preterm Birth Advisory Council meetings.

Themes	Subthemes	Description
**Research outcome priority**	Clinical Outcomes in Preterm Birth Research	Clinical outcomes and treatment efficacy are the top priority outcome in preterm birth research.
**Patient-led Care Model**	Preterm Birth Preventative care is a patient-led care model.	Psychological care a core aspect of management of pregnancy at high-risk of spontaneous preterm birth.
**Education around sPTB Risk Factors**	Awareness of risk factors for spontaneous preterm birth	Timely referral due to risk factor awareness improves patient experience and can improve outcomes.
**Preconceptual Counselling**	Reduction of trauma of unanticipated outcome	Anticipation of an event like preterm birth helps to reduce the trauma associated with an unexpected adverse outcome.
**Partner Inclusion**	Specific counselling and focus on partners	Partners are often overlooked in care in pregnancy at risk of spontaneous preterm birth.
**Language sensitivity**	Late mid-trimester loss	Referring to a pre-viability birth as a miscarriage can be extremely triggering and minimising for those affected.

### 1. Research outcome priority

Low priority was placed on the modality, route and duration of interventions to prevent preterm birth. The single greatest priority for women within preterm birth research outcomes, was the intervention efficacy for preterm birth prevention. Women described prevention of the preterm birth event as the most important factor when considering tolerability or acceptability of a treatment, and described high levels of tolerance for side effects. It was highlighted that adverse risks and side effects were important to include in discussions but had much lower likelihood of influencing decision-making if benefit on prevention efficacy was present.

“The most important outcome is a successful pregnancy. Any suggested procedures, any advice, guidance, trials, tests or medication made from the preterm surveillance clinic was welcomed and taken onboard!”

### 2. Patient-led care mode

Although clinical efficacy was identified as the top priority outcome, psychological support was identified as a core aspect of the experience of pregnancy at elevated risk of spontaneous preterm birth. Patient members did not place priority on the background of the care provider (obstetric versus midwifery) but rather the emphasis was their expertise in dealing with preterm birth prevention, sensitivity around prior history and continuity and support. In particular, the importance of counselling surrounding previous preterm birth experiences was highlighted as critical aspects of pregnancy care:

“Pregnancy following a preterm birth can bring a lot of traumatic thoughts. Personally, for me I knew as I was coming close to my previous pregnancy length of 30 weeks I was extremely upset and anxious. Overall, I can only see the benefits of counselling. If I was to provide guidance and advice to anyone else, it would be to do this as soon as possible.”

### 3. Education around sPTB risk factors

There was perception of inconsistency in the referral pathways to preterm birth prevention services. This lack of awareness was highlighted to exist in both the lay community and in primary and secondary level healthcare and maternity settings. There was also limited knowledge about available interventions to prevent preterm birth. Patient members identified this as a significant barrier for parents navigating pregnancy at risk of spontaneous preterm birth. Education around neonatal care was also highlighted as a potential support for those at high risk of spontaneous preterm birth:

“While I think it might be a difficult exercise to attempt to educate every pregnant person, I would like to see a little more documentation widely available from GPs, public health teams who can share a little on this. In terms of what could be shared during pregnancy, I would suggest a little information about what happens in a Neo-natal unit (in broad terms) and a little information on skin-to-skin, expressing milk, and the importance of reading and bonding during that time.”

### 4. Preconceptual counselling

Preconceptual counselling was highlighted as a critical component to the maternal experience of preterm birth prevention. Patient council members highlighted that anticipation of adverse events before pregnancy can improve sense of control, minimise trauma and increase ability to cope. The role of preconceptual counselling was highlighted by those with lived experience as a critical component of a preterm birth prevention service, and of high value to patients and their families.

“Having a plan can help you feel more in control and reduce the trauma of something unexpected happening in the next pregnancy.”

### 5. Partner inclusion

The partner’s experience of pregnancy at risk of preterm birth is largely missing from the focus of research and clinical care. Experts by experience highlighted that their partners lacked any specific supports, and their experience of spontaneous preterm birth was largely underappreciated. Peer-support, digital resources or partner handbooks were highlighted as helpful potential tools for support persons in pregnancy at risk of preterm birth:

“Partners and supportive friends/family members is really important. Making sure that partners know they are welcome to join appointments, any documents that can be specifically shared with partners they will take all of that on board. I would also suggest encouraging the partners to make sure they are encouraging a good diet, rest and counselling as regular reminders.”

### 6. Language sensitivity

Regarding language around preterm birth, there was no preferred term identified for women with risk factors or experience of preterm birth. However, patients referred to experiences of healthcare professionals using ‘miscarriage’ when referring to a mid-trimester pregnancy loss or peri-viability preterm birth.’ Labelling a late mid-pregnancy loss as a miscarriage was associated with significant trauma for patients. They highlighted that it minimises the loss, when in fact their experience is of birthing their baby. Improved sensitivity around the terminology of labour and loss in this late second trimester is critically important when caring for families who have experienced it.

“To this day, this phrase haunts me. I birthed my baby. I did not miscarry her.”

## Discussion

This paper describes themes within preterm birth research from focused discussion with those who have experienced preterm birth and the actions that these discussions heralded to improve patient care. The single greatest priority for women within preterm birth research outcomes, was the intervention efficacy for preterm birth prevention. Women described prevention of the preterm birth event as the most important factor when considering tolerability or acceptability of a treatment, and described high levels of tolerance for side effects. It was highlighted that adverse risks and side effects were important to include in discussions but had much lower likelihood of influencing decision-making if benefit on prevention efficacy was present. This perspective is crucial in validating the merit of large scale randomised trials ongoing to determine the efficacy of proposed interventions [13–15]. Clinical trials often require extensive resources in time, expertise and financial support. It is reassuring that these endeavours are critically important to the population they serve. These findings echo a previous study on priority core outcome sets for patients, where maternal and neonatal outcomes were higher priority than satisfaction, patient experience and health service use, and other patient related outcomes [8]. This prior study, a survey of 228 key stakeholders including parents and clinicians, also concluded clinical outcomes as highest priority in the research setting.

While medical and surgical interventions play their role in objectively reducing risk, much of pregnancy care after preterm birth centres on psychological support and continuity of care.

This support is mainly provided through continuity of care, patient familiarity with the service and regular engagement with prevention care providers. The healthcare provider best placed to provide this continuity is currently being explored [16]. Some studies have already demonstrated midwifery-led preterm birth prevention services have similar outcomes to obstetric-led services [17]. Adjuncts to preterm birth prevention care including risk scoring tools like the QUIPP app have also been found to alleviate maternal anxiety [18].

Awareness of risk factors for preterm birth is lacking in the lay population and among obstetric care providers, acting as a barrier to early pregnancy and pre-pregnancy referral to preterm birth prevention services. Risk factor awareness is critical for timely referral and effective prevention both during and in planning for pregnancy. This council highlights the need for effective education on risk factors for preterm birth, without insight, fear or increasing unnecessary anxiety or intervention. Group discussion identified that referral criteria for preterm birth prevention care should be highlighted through primary care physician integration groups, via their training bodies, using hospital social media platforms and integrating with cervical screening programs and colposcopy services.

Improved education and awareness is also critical to establish timely preconceptual referral for those at high-risk of preterm birth. The unexpected nature of adverse outcomes such as preterm birth adds additional trauma to the event itself. Patient members suggested those who have engaged in pre-pregnancy counselling can feel more empowered knowing how to engage in the service once pregnant, as well as familiarity with clinical care teams and expectations of preterm birth prevention aligned with evidence-based practice. Advanced planning from pre-pregnancy also allows maximal prevention strategies to be employed, including opportunity for interventions such as pre-pregnancy abdominal cerclage for those who need it. Patients members of this council highlight that preconceptual counselling has power to anticipate adverse outcomes, employ maximal preventative therapy but also ease the associated trauma for patients and their families.

The Advisory Council highlights that the paternal experience of spontaneous preterm birth and prevention is not well described or attended. Partner coping mechanisms differ from those of the mother, with male partners are often non-verbal after pregnancy loss [19], but there is very limited assessment of impact of preterm birth on the partner. Partner support during pregnancy is linked to improved maternal distress levels [20]. Thus adequate support and counselling for partners in pregnancy at risk of preterm birth has potential to both improve the experience of both parents and have impact on pregnancy outcome.

Strengths of this work include the collaborative partnership between patients and clinicians, fostered through the council meetings and collaborative endeavours. Patient members felt empowered at instigating action and improvements in clinical care. Clinicians felt renewed in enthusiasm for preterm birth prevention research including focus on the patient experience of preterm birth prevention. Personal experiences of preterm birth allowed rich data to be collected and analysed. Responses and views of patient members were unanimous across the majority of the themes identified. The limitations of a relatively small sample size are offset by the saturation of responses achieved prior to final participant response. The inclusion of seldom-heard patient voices in the preterm birth space is vital and under-reported.

## Conclusions

The Preterm Birth Advisory Council places the patient voice at the centre of preterm birth research. Efficacy of clinical intervention is the most important research outcome for patients, but adverse effects and side effects should also be described. Psychological support is of high priority to those embarking on pregnancy at risk of preterm birth, The experience and perspective of the partner voice is an urgent unmet focus with preterm birth research space. It is this council’s hope that through effective cocreation and partnership between patients and clinicians, it will be the patient’s voice that rings loudest through academic research in spontaneous preterm birth prevention.
